# Emerging Pyrethroid Resistance among *Anopheles arabiensis* in Kenya

**DOI:** 10.4269/ajtmh.17-0445

**Published:** 2018-01-22

**Authors:** Elizabeth Hemming-Schroeder, Stephanie Strahl, Eugene Yang, Amanda Nguyen, Eugenia Lo, Daibin Zhong, Harrysone Atieli, Andrew Githeko, Guiyun Yan

**Affiliations:** 1 Program in Public Health, University of California, Irvine, California;; 2 Centre for Vector Biology and Control Research, Kenya Medical Research Institute, Kisumu, Kenya

## Abstract

Vector control programs, particularly in the form of insecticide-treated bed nets (ITNs), are essential for achieving malaria elimination goals. Recent reports of increasing knockdown resistance (*kdr*) mutation frequencies for *Anopheles arabiensis* in Western Kenya heightens the concern on the future effectiveness of ITNs in Kenya. We examined resistance in *An. arabiensis* populations across Kenya through *kdr* mutations and World Health Organization–recommended bioassays. We detected two *kdr* alleles, L1014F and L1014S. *Kdr* mutations were found in five of the 11 study sites, with mutation frequencies ranging from 3% to 63%. In two Western Kenya populations, the *kdr* L1014F allele frequency was as high as 10%. The L1014S frequency was highest at Chulaimbo at 55%. Notably, the *kdr* L1014F mutation was found to be associated with pyrethroid resistance at Port Victoria, but *kdr* mutations were not significantly associated with resistance at Chulaimbo, which had the highest *kdr* mutation frequency among all sites. This study demonstrated the emerging pyrethroid resistance in *An. arabiensis* and that pyrethroid resistance may be related to *kdr* mutations. Resistance monitoring and management are urgently needed for this species in Kenya where resistance is emerging and its abundance is becoming predominant. *Kdr* mutations may serve as a biomarker for pyrethroid resistance in *An. arabiensis*.

## INTRODUCTION

Despite intensive malaria control efforts, malaria remains a leading cause of morbidity and mortality in Kenya, especially among younger children and pregnant women.^1^ Vector control programs, particularly in the form of insecticide-treated bed nets (ITNs) are essential for achieving malaria elimination goals^2,3^ and have coincided with a decrease in malaria-related morbidity rates in Kenya.^4^ However, increasing insecticide resistance threatens the efficacy of antimalarial interventions.^5^

Pyrethroids are the only approved insecticide for use in ITNs.^6^ Its low mammalian toxicity and induction of paralysis using nerve stimulation of dysfunctional sodium channels makes it ideal for ITN usage.^5,7^ However, a single amino acid change at residue position 1014 in the voltage-gated sodium channel (VGSC) gene of insects has made the insecticide increasingly obsolete. This mutation has been shown to confer knockdown resistance (*kdr*) by decreasing sodium channel affinity for the insecticide binding site.^8^ The *kdr* mutations are found as L1014F (*kdr*-west) and L1014S (*kdr*-east) in *Anopheles gambiae*.^9^ L1014F refers to a point mutation from leucine to phenylalanine, whereas L1014S represents a mutation from leucine to serine.^9,10^ Originally, L1014F was found in Western Africa, hence leading to its name *kdr*-west,^11–14^ whereas L1014S (*kdr*-east) was found in Eastern Africa.^10,15^ However, both mutations are now found throughout Africa and have not been solely concentrated geographically, thus suggesting a shift in *kdr* mutation frequencies in endemic countries.^16–20^ In addition, both *kdr* mutations have been associated with increased susceptibility to *Plasmodium falciparum*, further heightening malaria risk in areas with high insecticide resistance.^21^

Mass distribution of ITNs has been followed by a rapid increase in *kdr* alleles and insecticide resistance in *An. gambiae* s.s.^5^ In Kenya, where ITN coverage increased from less than 10% in 2004^22^ to greater than 80% since 2013,^23^ *kdr* mutation frequencies in *An. gambiae* s.s. increased rapidly from 6% in 2001^15^ to near fixation at 98% in 2010.^5^ In addition to the rise of *kdr* mutation frequencies in *An. gambiae* s.s., higher ITN usage has led to a species shift from primarily *An. gambiae* s.s. to *Anopheles arabiensis*.^2,24–27^ As such, the contribution of *An. arabiensis* to malaria transmission increases in malaria-endemic areas under the current ITN program.

Recently, *kdr* mutation frequencies in *An. arabiensis* from Western Kenya have been found to be increasing and were as high as 13% and 39% at certain localities in 2013.^6,23^ Previously, in 2005, *kdr* mutation frequencies were not found to exceed 6% at any locality in Western Kenya^28^ and, moreover, were not detected in 2009.^29^ Although the evasion of ITNs might explain why the frequency of *kdr* mutations and physiological insecticide resistance in *An. arabiensis* has remained relatively low with respect to *An. gambiae* s.s., we expect an increase in *kdr* mutations for *An. arabiensis* to continue. However, we do not expect *kdr* mutations to increase as rapidly in *An. arabiensis* as they did in *An. gambiae* s.s. because of the reduced selection pressure imposed on *An. arabiensis* which more commonly feed outdoors.

Although ITNs are presently the most cost-effective method of preventing malaria, increased insecticide resistance, and outdoor biting reduce their efficacy and present a major threat to malaria control programs.^1^ Previous studies have examined the spatial distribution of *kdr* mutations in various *An. arabiensis* populations in Africa,^5–7,12^ but the association between *kdr* mutations and phenotypic resistance is not well established. Therefore, this study aimed to examine the link between *kdr* mutations and pyrethroid resistance by comparing the genotypes of phenotypically resistant and susceptible mosquitoes.

## MATERIALS AND METHODS

### Study design for *kdr* survey.

**Figure 1.**
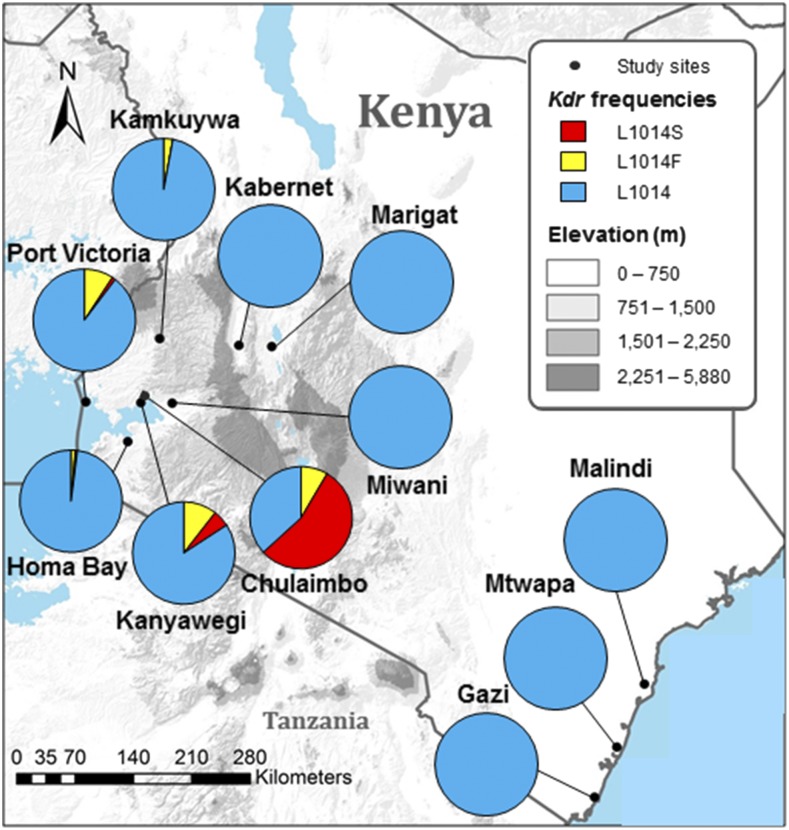
Knockdown resistance (*kdr*) allele frequencies in *Anopheles arabiensis* populations across Kenya, 2014. 1014F mutation prevalences: Kanyawegi (10.5%), Port Victoria (9.2%), Chulaimbo (8.5%), Kamkuywa (2.9%), Homa Bay (1.7%), Kabernet (0.0%), Marigat (0.0%), Miwani (0.0%), Gazi (0.0%), Mtwapa (0.0%), and Malindi (0.0%). 1014S mutation prevalences: Chulaimbo (54.7%), Port Victoria (1.1%), Homa Bay (0.6%), Kanyawegi (5.3%), Kamkuywa (0.0%), Kabernet (0.0%), Marigat (0.0%), Miwani (0.0%), Gazi (0.0%), Mtwapa (0.0%), and Malindi (0.0%). This figure appears in color at www.ajtmh.org.

*Anopheles gambiae* s.l. larvae were collected from 11 study sites across Kenya between May 2014 and October 2014 (Figure 1). Not more than five larvae were collected from a given habitat to reduce sampling bias. Sampling bias was tested by comparing mutation frequencies to frequencies when randomly selecting one larva per habitat, and no significant differences were found. Study sites were selected across the diverse geographical regions of Kenya. The major regions were the lowlands surrounding Lake Victoria in Western Kenya (Port Victoria, Homa Bay, Kanyawegi, Chulaimbo, and Miwani), the highlands in Western Kenya (Kamkuywa), the Great Rift Valley in Western Kenya (Kabernet and Marigat), and coastal Kenya (Malindi, Mtwapa, and Gazi).

### World Health Organization (WHO) bioassays.

To explore the link between *kdr* mutations and pyrethroid resistance, we genotyped phenotypically resistant and susceptible *An. arabiensis*, determined by a standard WHO insecticide susceptibility bioassay.^30^ *Anopheles gambiae* s.l. larvae were collected from Port Victoria and Chulaimbo, study sites where *kdr* mutations in *An. arabiensis* had previously been detected,^22^ and reared to adults. Adult female mosquitoes 2–3 days old were aspirated into exposure tubes in batches of 15–20 mosquitoes per tube. Tubes were lined with insecticide (0.05% deltamethrin)-impregnated paper. A subset of tubes was only lined with oil paper to serve as controls. In addition, the Kisumu-susceptible *An. gambiae* s.s. strain was used as a control. After being held in their respective tubes for 60 minutes, mosquitoes were transferred to a holding tube with 10% sucrose solution and put to standard insectary conditions for 24 hours. These mosquitoes were screened again. If after 24 hours, mosquitoes were knocked down such that they were either dead or unable to fly, they were classified as susceptible.

### Procedures.

Genomic DNA was extracted from individual mosquitoes using standard ethanol extraction procedures with phenol:chloroform.^31^ The final DNA pellet was suspended in 20 μL of 10 mM Tris and 1 mM EDTA buffer. A NanoDrop 1000 Spectrophotometer was used to quantify DNA concentrations, and stock DNA was diluted to an approximate concentration of 1 μg/μL for use in polymerase chain reaction (PCR). *Anopheles arabiensis* and *An. gambiae* s.s. were identified within the *An. gambiae* s.l. complex using a ribosomal DNA PCR assay.^32^ We genotyped 683 *An. arabiensis* for *kdr* alleles: L1014 (wild-type), L1014F (*kdr*-west), and L1014S (*kdr*-east) using a Taqman probe assay.^33^ For detection, the wild-type alleles were labeled with 4,7,2′-trichloro-7′-phenyl-6-carboxyfluorescein at the 5′ end and the 1014F and 1014S *kdr* alleles were labeled with 6-carboxyfluorescein.

### Statistical analysis.

For the WHO bioassay, Fischer’s exact tests were performed to make pairwise comparisons for mutation frequencies between resistant and susceptible groups. Odds ratios (ORs) were used to quantify the association between *kdr* genotype and insecticide-resistant phenotype. Chulaimbo and Port Victoria populations were analyzed separately.

## RESULTS

### *Kdr* survey.

A total of 1,425 *An. gambiae* s.l. specimens were examined (Table 1). *Anopheles arabiensis* proportions ranged from 12.8% at Chulaimbo to 100% at Miwani, Bogoria, Gazi, Mtwapa, and Malindi (Table 1). *Kdr* mutations were detected in five *An. arabiensis* populations: Port Victoria (10.3%), Homa Bay (2.3%), Kamkuywa (2.8%), Kanyawegi (15.8%), and Chulaimbo (63.2%) (Figure 1). The 1014F mutation prevalence was highest at Port Victoria (9.2%), Kanyawegi (10.5%), and Chulaimbo (8.5%), but also observed at Kamkuywa (2.9%) and Homa Bay (1.7%). The 1014S mutation was prevalent at Chulaimbo (54.7%) and detected at low frequencies at Port Victoria (1.1%), Homa Bay (0.6%), and Kanyawegi (5.3%). No mutations were observed in populations outside Western Kenya. The population at Chulaimbo was the only population that significantly deviated from the Hardy–Weinberg equilibrium with regard to *kdr* alleles (Table 1).

**Table 1:** Proportion of *Anopheles arabiensis* within the *Anopheles gambiae* s.l. species complex and knockdown resistance genotype frequencies with the Hardy–Weinberg equilibrium parameters for *An. arabiensis* collected in Kenya, 2014

Site	Elevation	Number	*An. arabiensis* (%)	Genotype frequencies (%)*					Hardy–Weinberg equilibrium	
				LL	LF	FF	LS	SS	*H*_E_†	*F*_IS_‡
Port Victoria	1,139	168	56.5	80.4	18.5	0.0	0.0	0.0	0.187	0.013
Homa Bay	1,184	133	68.4	95.3	3.5	0.0	1.2	0.0	0.046	−0.019
Kamkuywa	1,487	72	52.8	91.9	5.4	0.0	0.0	0.0	0.054	0.000
Kanyawegi	1,214	129	47.3	71.1	15.8	2.6	10.5	0.0	0.028	0.050
Chulaimbo	1,377	446	12.8	26.9	17.3	0.0	0.0	55.8	0.558	0.690§
Miwani	1,161	120	100	100	0.0	0.0	0.0	0.0	0.000	–
Marigat	1,004	94	100	100	0.0	0.0	0.0	0.0	0.000	–
Kabernet	1,150	101	92.1	100	0.0	0.0	0.0	0.0	0.000	–
Gazi	15	30	100	100	0.0	0.0	0.0	0.0	0.000	–
Mtwapa	66	44	100	100	0.0	0.0	0.0	0.0	0.000	–
Malindi	14	88	100	100	0.0	0.0	0.0	0.0	0.000	–

* L is wild-type at L1014 codon; F is L1014F mutation; S is L1014S mutation.

† *H*_E_ expected heterozygosity.

‡ *F*_IS_ inbreeding coefficient.

§ Significant deviation from the Hardy–Weinberg equilibrium.

### WHO bioassay.

**Figure 2.**
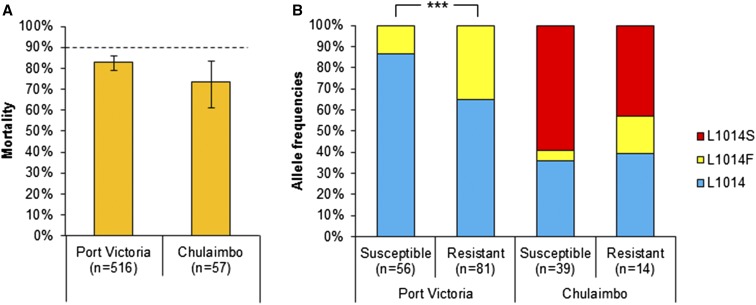
Mortality rates (**A**) and frequencies of knockdown resistance alleles of susceptible and resistant groups (**B**) in *Anopheles arabiensis* populations in Kenya. The dotted line indicates World Health Organization threshold for confirmed resistance (90%). *** indicates *P* < 0.001. Error bars indicate 95% confidence interval (CI). Mortality rates at Port Victoria: 82.8% (95% CI = [0.792–0.859]) and Chulaimbo: 73.7% (95% CI = [0.610–0.834]). 1014F mutation prevalences: Port Victoria Susceptible (13.3%), Port Victoria Resistant (35.2%), Chulaimbo Susceptible (5.1%), and Chulaimbo Resistant (17.9%). 1014S mutation prevalences: Port Victoria Susceptible (0.0%), Port Victoria Resistant (0.0%), Chulaimbo Susceptible (59.0%), and Chulaimbo Resistant (42.9%). This figure appears in color at www.ajtmh.org.

The control Kisumu-susceptible *An. gambiae* s.s. strain had a mortality rate of 100%. We observed a mortality rate of 82.8% (95% confidence interval [CI] = [0.792–0.859]) and 73.7% (95% CI = [0.610–0.834]) for *An. arabiensis* at Port Victoria and Chulaimbo, respectively. Both mortality rates were lower than the WHO 90% threshold for resistance (Figure 2A).

A comparison of *kdr* mutation frequencies between a subset of resistant and susceptible *An. arabiensis* revealed that deltamethrin-resistant mosquitoes had significantly higher frequencies of the L1014F mutation at Port Victoria (OR = 3.495, 95% CI = [1.809–7.102], *P* < 0.001, Fischer’s exact test) (Figure 2B), supporting the link between the *kdr* mutation and pyrethroid resistance. Although both L1014F and L1014S mutations were detected at Chulaimbo, the highest resistant field population, there was no significant difference in allele frequencies between susceptible and resistant groups (*P* = 0.078; Fischer’s exact test) (Figure 2B). When comparing only the L1014F frequency between groups at Chulaimbo, the difference is marginally significant (OR = 3.957, 95% CI = [0.781–21.713], *P* = 0.053; Fischer’s exact test) and could be limited by a low sample size in the resistance group (*N* = 14), whereas there was no significant difference in L1014S frequencies between susceptible and resistant groups (OR = 0.525, 95% CI = [0.197–1.364], *P* = 0.185, Fischer’s exact test).

## DISCUSSION

The observed high proportions of *An. arabiensis* in this study demonstrate the ongoing species composition shift from predominantly *An. gambiae* s.s. to *An. arabiensis* in East Africa.^2,24–27^ A decline in *An. gambiae* s.s. relative abundance yet stable population of *An. arabiensis* has been observed in the lowlands of Kenya in conjunction with an increase in ITN coverage.^2,7,23,27^ These findings underscore the importance of the role that *An. arabiensis* are playing in maintaining residual malaria transmission, and as such, will present a major barrier to malaria control and elimination. Understanding *An. arabiensis* insecticide resistance mechanisms and monitoring for resistance are essential for achieving malaria elimination goals.

The presence of *kdr* mutations at several sites in Western Kenya indicates the widespread occurrence of *kdr* mutations among *An. arabiensis* populations. In particular, the L1014F mutation, first detected in Kenya in 2012,^6^ was observed in four of the five Western Kenya populations in this study. The emergence of L1014F was also found in neighboring malaria-endemic countries. L1014F has recently been detected in Tanzania in both *An. gambiae* and *An. arabiensis* populations.^34^ Moreover, high frequencies of the L1014F mutation in *An. arabiensis* have been reported from Ethiopia^35–37^ and central Sudan.^38^ A continual increase in this mutation prevalence in Kenya may cause further concern on the future utility of ITNs.

The rise of the L1014F mutation may be particularly concerning, given that this mutation was found to be associated with pyrethroid resistance in *An. arabiensis* in our Port Victoria study population. *Kdr* mutations at Chulaimbo were not significantly associated with pyrethroid resistance. This result could be due to the low frequency of L1014F and presence of the L1014S mutation at this site. The prevalence in L1014F mutations was higher in the resistant group at Chulaimbo, but the difference was not statistically significant. In *An. gambiae* s.s., the L1014S mutation has been found to be more weakly associated with pyrethroid resistance than the L1014F mutation.^39^ Similarly, the L1014F mutation may also have a stronger association with pyrethroid resistance in *An. arabiensis*. In Sudan, there was also a significant association found between the 1014F mutation and DDT and pyrethroid resistance in *An. arabiensis*, but the 1014S mutation was not detected in the populations tested.^38^ Further studies are needed to investigate the role of the 1014S and 104F mutations in *An. arabiensis* insecticide resistance. The result also suggests that other mechanisms such as metabolic detoxification or secondary mutations at alternative loci could be involved in pyrethroid resistance in *An. arabiensis* at Chulaimbo, especially given the high levels of resistance at this site. Metabolic resistance using rapid insecticide detoxification due to the overexpression of P450 enzymes has been found to be a common resistance mechanism for *An. arabiensis*.^35,40–42^

Interestingly, *kdr* mutations were only observed in *An. arabiensis* specimens from study sites where *An. gambiae* were also common at proportions exceeding 30%. Stump et al.^15^ first suggested the possibility that *kdr* alleles could have been introduced into Kenyan *An. arabiensis* populations through introgression. Adaptive introgression of *kdr* alleles has been supported by evidence of consequential contemporary gene flow between *An. arabiensis* and *An. gambiae* in East Africa.^43,44^ This notion is underscored by findings of identical intron sequences in the VGSC between the two species in Kenya.^29^ Our findings of *kdr* mutations occurring exclusively in *An. arabiensis* populations where *An. gambiae* are common are consistent with the hypothesis that *An. arabiensis* acquire *kdr* mutations through introgression with sympatric *An. gambiae* populations.

Pyrethroid resistance in *An. arabiensis* has been reported in several countries, including Sudan,^38^ Ethiopia,^35,45^ Malawi,^46^ Tanzania,^47^ Zanzibar,^48,49^ and Kenya.^7^ Despite widespread resistance in major malaria vectors in sub-Saharan Africa, pyrethroids are the only approved insecticide for use in ITNs.^6^ The findings from this study and Abdalla et al.^38^ that the L1014F mutation is associated with pyrethroid resistance in *An. arabiensis* provide evidence on the utility of screening *An. arabiensis* populations for *kdr* mutations in informing pyrethroid resistance status and trends. However, that *kdr* mutations were not associated with resistance at Chulaimbo also highlights the complexity of insecticide resistance and the need for further studies on resistance mechanisms in *An. arabiensis*.

*Kdr* mutations could potentially increase and spread rapidly in a pattern like that observed for *An. gambiae* from 2001 to 2010.^5,15^ Our results of commonly occurring 1014F mutations associated with pyrethroid resistance in *An. arabiensis* underscores the importance in searching for alternative methods to pyrethroid-impregnated bed nets for vector control. High levels of resistance in *An. gambiae* s.s.,^5^ *An. arabiensis* behavioral resistance to ITNs,^2^ an increased proportion of *An. arabiensis*, and frequent *kdr* mutations in *An. arabiensis* from Western Kenya could all contribute to compromised efficacy of ITNs. Therefore, complementary interventions targeting outdoor mosquitoes, such as attractive toxic sugar–baited traps, habitat reduction, and/or biological larvicides, could be important to improving the overall efficacy of antimalarial programs, as well as suppressing pyrethroid resistance. These interventions have been effective for vector control in areas such as Mali,^50^ Ecuador,^51^ Peru,^51^ and Kenya.^52^

In summary, we found evidence of widespread *kdr* mutations in Western Kenya and an association between the *kdr* 1014F mutation and pyrethroid resistance in *An. arabiensis*. This result is concerning for the effectiveness of ITNs, especially because *An. arabiensis* is becoming the predominant malaria vector in Kenya and throughout Africa.^2^ Monitoring for the spread of insecticide resistance in *An. arabiensis* is critical for resistance management, and consequently, the success of vector control programs.
